# Thermal conductivity enhancement in thermal grease containing different CuO structures

**DOI:** 10.1186/s11671-015-0822-6

**Published:** 2015-03-08

**Authors:** Wei Yu, Junchang Zhao, Mingzhu Wang, Yiheng Hu, Lifei Chen, Huaqing Xie

**Affiliations:** College of Engineering, Shanghai Second Polytechnic University, 2360 Jin Hai Road, Pudong District,, Shanghai, 201209 China; Shanghai Yueda New Materials Science and Technology Ltd., 2588 Jin Hai Road, Pudong District, Shanghai, 201209 China

**Keywords:** CuO structures, Thermal conductivity, Large aspect ratio, Thermal grease

## Abstract

Different cupric oxide (CuO) structures have attracted intensive interest because of their promising applications in various fields. In this study, three kinds of CuO structures, namely, CuO microdisks, CuO nanoblocks, and CuO microspheres, are synthesized by solution-based synthetic methods. The morphologies and crystal structures of these CuO structures are characterized by field-emission scanning electron microscope and X-ray diffractometer, respectively. They are used as thermal conductive fillers to prepare silicone-based thermal greases, giving rise to great enhancement in thermal conductivity. Compared with pure silicone base, the thermal conductivities of thermal greases with CuO microdisks, CuO nanoblocks, and CuO microspheres are 0.283, 0256, and 0.239 W/mK, respectively, at filler loading of 9 vol.%, which increases 139%, 116%, and 99%, respectively. These thermal greases present a slight descendent tendency in thermal conductivity at elevated temperatures. These experimental data are compared with Nan's model prediction, indicating that the shape factor has a great influence on thermal conductivity improvement of thermal greases with different CuO structures. Meanwhile, due to large aspect ratio of CuO microdisks, they can form thermal networks more effectively than the other two structures, resulting in higher thermal conductivity enhancement.

## Background

Different cupric oxide (CuO) structures with unique morphology possess superior physical and chemical properties that remarkably differ from those of their bulk counterparts due to the size effects [1]. These structures have attracted intensive interest because of their promising applications in various fields [1]. In light of the previous results of researchers around the world, the physical and chemical properties of the nanostructured CuO are closely related to morphologies and sizes of CuO structures [2-6]. In addition, the development of synthetic methods has been widely accepted as an area of fundamental importance to the understanding and application of nanoscale materials [1]. Consequently, numerous methods have been recently developed to synthesize various CuO structures with diverse morphologies, sizes, and dimensions using various chemical and physical strategies. Generally speaking, solution-based synthetic methods are very common and effective ways to prepare various CuO structures with good control of shape, composition, and reproducibility [7-9]. Among various solution-based synthetic methods, hydrothermal and chemical precipitation techniques have been widely used in synthesizing CuO structures that benefited from the entire process which can be rationally and precisely controlled [7,10-13]. In this paper, such methods are selected to synthesize CuO structures.

Typically, metal oxides have lower heat transfer capacities than corresponding metals with few exceptions such as CuO with a reasonable thermal conductivity [14]. CuO particles, hence, have provided a promising place in thermal conductivity enhancement of composites [15] and nanofluids [16-18]. The studies [16-18] showed that the CuO particles could be effective fillers for thermal conductivity improvement in nanofluid systems. According to the theories based on effective medium theory of thermal conductivity of composite, the size, shape, and thermal conductivity of filler have a great influence on thermal conductivity enhancement of polymer composites and nanofluids [19,20], but the influence of the shape of CuO particles on thermal conductivity improvement in thermal grease system has been overlooked.

Meanwhile, as far as we know, the enhanced thermal conductivity of thermal grease with CuO particles has not been reported. Thermal grease is a commonly used thermal interfacial material for heat removal from electronic devices [21]. It is utilized to bond the contact surfaces of heat sink and devices and to replace the air in the gaps at the interface, thus establishing an effective thermal path between a heat-generating component and a heat sink attached to it [22,23]. Therefore, in this study, three kinds of CuO with different shapes, namely, CuO microdisks, CuO nanoblocks, and CuO microspheres, were synthesized by solution-based synthetic methods. They were used to prepare silicone-based thermal greases, and influences of their shape and size on thermal conductivity improvement were investigated. The obtained results were also compared with theoretical prediction model.

## Methods

### Synthesis of different CuO structures

Reagents, purchased from Sinopharm Chemical Reagent Co., Ltd., Shanghai, China, were analytical grade and used without further purification unless specified. The CuO microdisks were synthesized as follows: 0.998 g CuSO_4_∙5H_2_O was dissolved in 100 mL distilled water stirred with a magnetic stirrer for 15 min to ensure that the CuSO_4_ dissolved completely. Then, 30 mL of 0.15 M NH_3_∙H_2_O was quickly added into the CuSO_4_ solution under constant stirring. After stirring of 15 min, 6 mL of 1.2 M NaOH was added dropwise into the above solution under constant stirring until a blue precipitate was produced. The solution containing blue precipitate was heated at 80°C for 5 h to ensure that the blue precipitate changed to the dark brown CuO completely.

The CuO nanoblocks were synthesized by hydrothermal method as follows: a copper precursor solution (0.2 M) was prepared in a beaker by addition of Cu(NO_3_)_2_∙3H_2_O in distilled water. The solution was mixed with NaOH (0.2 M) solution under continuous stirring, and then it was transferred into a Teflon-lined stainless steel autoclave. The autoclave was maintained at a temperature of 180°C for 24 h and then cooled to atmospheric temperature; a black precipitate was obtained.

The CuO microspheres were synthesized according to the method reported by Jia *et al.* [24] with some modification. A solution containing 0.015 M copper acetate and 0.015 M urea was placed in Teflon-lined stainless steel autoclave and maintained at 120°C for 5 h. The autoclave was cooled to room temperature and a black precipitate was obtained.

All the three black CuO precipitates were washed with distilled water several times, filtered, and dried in an oven at 80°C for 12 h.

### Preparation of thermal grease

The thermal greases with different CuO structures were prepared as follows: for example, the CuO microdisks with different volume fractions were mixed with the silicone base by using a planetary mixer/dearator (Mazerustar KK-250S, Kurabo, Osaka, Japan) for 30 min at room temperature, and the thermal grease with different loading CuO microdisks was obtained. The thermal greases with different loadings of CuO nanoblocks and microspheres were prepared by the same procedure.

### Characterization

The morphology of the CuO structures was observed by field-emission scanning electron microscope (SEM) (S4800, Hitachi, Tokyo, Japan). The crystal structure of the samples was characterized by X-ray diffractometer (XRD) (D8 Advance, Bruker, Karlsruhe, Germany) equipped with a copper target and nickel filter. X-ray wavelength used in the analysis was 0.154 nm of CuKa. The thermal conductivities of the composites were measured by a thermal conductivity analyzer (C-Therm TCi, C-Therm Technologies Ltd., Fredericton, Canada), which is based upon the modified transient plane source principle. The TCi system consists of a sensor, power control device, and computer software. A spiral-type heating source is located at the center of the sensor, and heat is generated at the center. The heat that has been generated enters the material through the sensor during which a voltage decrease occurs rapidly at the heating source, and the thermal conductivity is calculated through the voltage decrease data. The testing capabilities of the system are 0 to 100 W/mK across a wide range of temperature (−50°C to 200°C). For this measurement, the samples were filled into the mold with a thickness of 2 mm. The uncertainty of this test method is estimated to be within ±1.0%, which is decided by the test instrument. The thermal conductivity of each sample is tested five times to obtain the average value. The temperature of test system was controlled by constant temperature box (Shanghai Boxun Industry & Commerce Co., Ltd., Shanghai, China).

## Results and discussion

### Morphology of CuO structures

**Figure 1:**
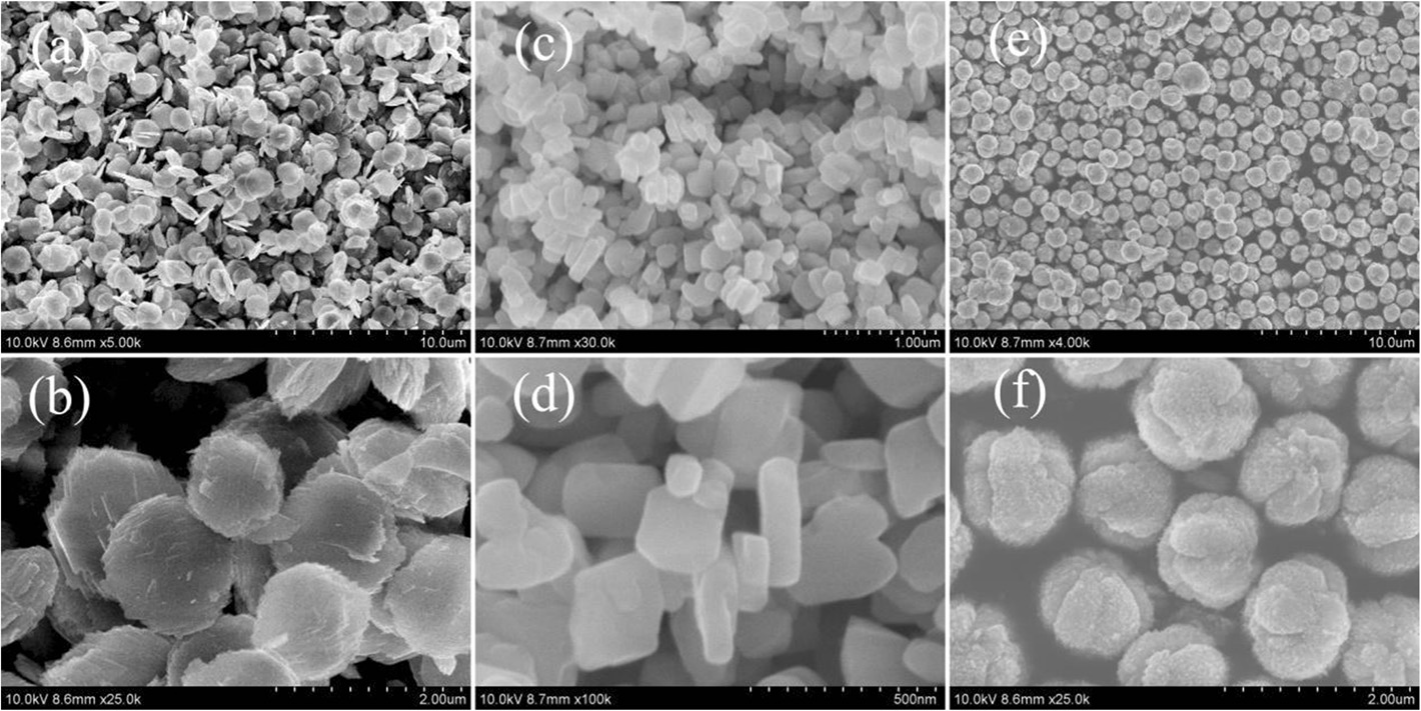
**FE-SEM images of different structures. (a)** CuO microdisks, **(c)** CuO nanoblocks, and **(e)** CuO microspheres at low magnification; **(b)** CuO microdisks, **(d)** CuO nanoblocks, and **(f)** CuO microspheres at high magnification.

The morphologies of as-synthesized CuO structures were observed by using field-emission scanning electron microscope, shown in Figure 1. Figure 1a is the low-magnification SEM image of as-synthesized CuO structures, confirming that the products are synthesized nearly with same morphology. The synthesized products are round or hexagonal disks of CuO as revealed by the high-magnification SEM image presented in Figure 1b. The average planar sizes of CuO microdisks are in range of 1.0 ~ 1.8 μm. The thicknesses of the CuO microdisks are in range of 50 ~ 100 nm, which is obtained from the analysis of SEM images with right position. Meanwhile, the surfaces of CuO microdisks are not smooth, and some small nanosheets are horizontally deposited on the upper surface of the microdisks, which suggests that the microdisks are formed by the accumulation of CuO nanosheets. Seen from the Figure 1c,d, the as-synthesized CuO structures are nanoblocks with planar size of 200 ~ 350 nm and thickness of 100 ~ 150 nm. The surfaces of the nanoblocks are very smooth. Figure 1e,d shows the FE-SEM images of CuO products at low and high magnification, respectively. The as-synthesized CuO structures are microspheres with diameter of about 1 μm. The surface of the microspheres is rough with some gullies.

### XRD pattern of CuO structures

**Figure 2:**
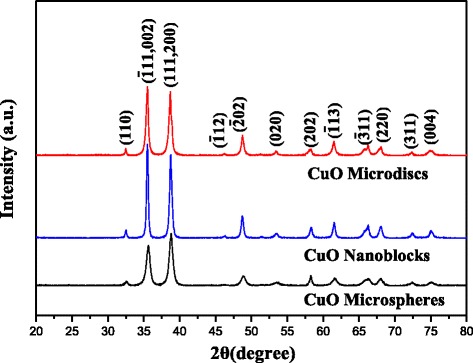
**Typical XRD patterns of different CuO structures.**

The crystallinity and crystal phases of the synthesized structures were analyzed by the X-ray diffraction pattern shown in Figure 2. It is seen from the XRD pattern of CuO microdisks that all of the diffraction peaks can be clearly indexed to the monoclinic structured CuO with lattice constants of *a* = 0.4684 nm, *b* = 0.3425 nm, *c* = 0.5129 nm, and *β* = 99.54° from the standard card JCPDS 05-0661. The diffraction peaks can be readily indexed as (110), ($\overline{1}$ 11,002), (111,200), ($\overline{1}$ 12), ($\overline{2}$ 02), (020), (202), ($\overline{1}$ 13), ($\overline{3}$ 11), (220), (311), and (004) planes of CuO with a monoclinic structure, respectively. Importantly, no peak related to any by-products such as Cu(OH)_2_, Cu_2_O, or Cu are seen in the observed pattern, which confirms that the as-synthesized products are pure CuO. The peak intensities and widths clearly indicate that the sample is highly crystalline in nature. The XRD patterns of CuO nanoblocks and CuO microspheres present the same diffraction peaks as CuO microdisks, which states clearly the monoclinic structured crystalline of them as well.

### Thermal conductive properties of different thermal greases

**Figure 3:**
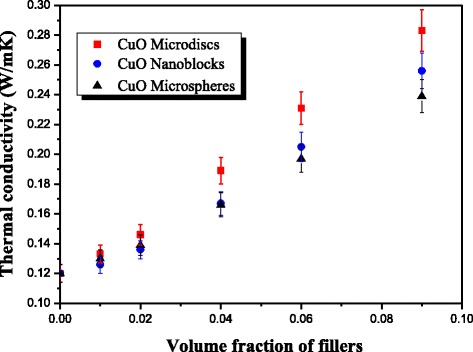
**Thermal conductivity of thermal grease with different filler loadings.**

The thermal conductivity of different thermal greases containing different CuO structures is shown in Figure 3. It can be seen that the thermal conductivity of the different greases is improved greatly compared with silicone base. The thermal conductivity of the three greases based on different CuO structures increase with the increase of volume fraction of fillers. The maximum volume fraction of the three fillers in this study is 9%, because beyond this value, the viscosity of these composites becomes very large and lost its mobility at room temperature. It can be seen from Figure 3 that the thermal conductivity of thermal grease with these three kinds of CuO particles increases slowly and are almost equal when the volume fraction is less than 4%. At low filler loading, the heat-conductive CuO particles surrounded by matrix cannot touch each other; hence, the thermal conductivity increases very slowly resulting from high thermal contact resistance inside the composites [25]. The size and shape do not obviously affect the thermal conductivity of thermal greases either. While with the loading further increasing, clearly seen from Figure 3, the thermal conductivity of grease with different CuO differs greatly, which indicates the shape and size of CuO structures have a significant influence on the thermal conductivity improvement of thermal grease. At the same volume fraction, the thermal conductivity of thermal grease with CuO microdisks is larger than that with CuO nanoblocks and CuO microspheres. The thermal conductivities of thermal greases with CuO microdisks, CuO nanoblocks, and CuO microspheres are 0.283, 0256, and 0.239 W/mK, respectively, at filler loading of 9 vol.%, which increases 139%, 116% and 99%, respectively, compared with pure silicone base (0.12 W/mK). It is worth noting that the thermal conductivity enhancements of thermal greases with CuO microdisks, CuO nanoblocks, and CuO microspheres are 57%, 40%, and 39%, respectively, with a loading of 4 vol.%. They are higher than the values reported by Liu *et al.* [16] and Zhu *et al.* [17] for nanofluids with CuO nanoparticles. It suggests that CuO microdisks are more effective fillers in contributing to thermal conductivity improvement than CuO nanoblocks and CuO microspheres. The main reason is that CuO microdisks used in this research with a large aspect ratio can easily contact with each other and thus to structure some effective thermal conductive networks. These networks can facilitate phonon transfer in the grease, resulting in high enhancement in thermal conductivity [26,27]. Moreover, the thermal conductivity of thermal grease with nanosized CuO nanoblocks is higher than that of thermal grease with microsized CuO microspheres. The larger particle size is desired to minimize the scattering of phonons because of low interfacial thermal barrier [28,29]. We speculate that CuO nanoblocks will aggregate to form clusters due to the nanosize effect of CuO nanoblocks when the loading is high. These clusters with relatively large size could form some effective thermal conductive networks, thus to cause the thermal conductivity enhancement [30], which is the cause of this anomaly.

**Figure 4:**
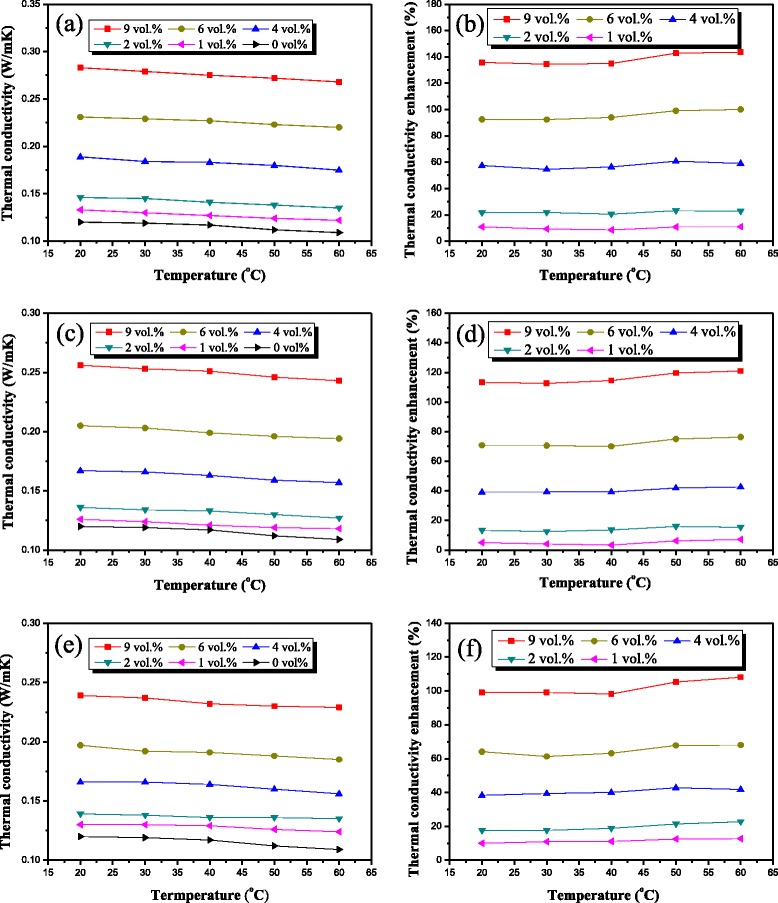
**Thermal conductivity and thermal conductivity enhancement of thermal greases.** Thermal conductivity and thermal conductivity enhancement were based on **(a)**, **(b)** CuO microdisks; **(c)**, **(d)** CuO nanoblocks; and **(e)**, **(f)** CuO microspheres with tested temperatures varying.

Although some groups have reported studies of the thermal conductivity enhancement at elevated temperatures, there are relatively fewer effective data to reach the unanimous conclusion about the influence of temperature on thermal conductivity. In this paper, the effect of temperature on thermal conductivity of thermal greases was investigated by measuring the thermal conductivity of thermal greases for different temperatures ranging from 20°C to 70°C, as shown in Figure 4. Figure 4a shows a continuous decrease of thermal conductivity of all the different thermal greases with CuO microdisks at elevated temperatures. Meanwhile, the thermal conductivity results at different temperatures varying the particle concentration for CuO nanoblocks and CuO microspheres follow the same trend as CuO microdisks, as shown in Figure 4c,e. In order to study the separate role of CuO particles for thermal conductivity improvement, the thermal conductivity enhancements of thermal greases are calculated with respect to that of pure silicone at the corresponding temperature, as shown in Figure 4b,d,f. The thermal conductivity enhancement tendency of these three thermal greases is almost constant at elevated temperature. This phenomenon is in contrast to the reported results about nanofluid with CuO [14,18]. In their experimental system, the low viscosity base fluids and nanosized CuO particles were used. Therefore, Brownian motions of the suspended nanoparticles will become more intensive when the temperature is ascended. The micro-convection caused by the Brownian motions would help to enhance the thermal conductivity of the suspensions. However, because of high viscosity of the silicone base and relatively large size of CuO particles, the effect of Brownian motions is not so obvious [31]. Together with the downward trend of thermal conductivity of silicone base at elevated temperatures, a slight descendent tendency is caused.

### Comparison to theoretical predictive models

To further investigate the enhancement of the thermal conductivity of thermal grease with different CuO structures, the experimental results (at 20°C) are compared with the theoretical values predicted by Nan's model equation [32,33]:

1 $$ \frac{k_c}{k_m}=1+\frac{\varphi p}{3}\frac{k_f/{k}_m}{p+\frac{2{a}_K{k}_f}{d{k}_m}} $$

where *φ* is the volume fraction of the fillers, *k*_c_ is the thermal conductivity of the thermal grease, *k*_m_ is the thermal conductivity of the silicone, *k*_f_ is the thermal conductivity of filler, *a*_K_ is the Kapitza radius (*R*_k_ × *k*_m_, *R*_k_ is the interface thermal resistance between the filler and matrix), *p* is the shape factor, whereby it is the aspect ratio of the filler, and *d* is the diameter or thickness of the filler.

**Figure 5:**
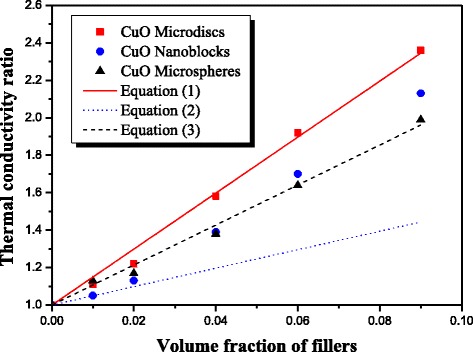
**Comparison of theoretical predictive model with experimental data.**

Figure 5 shows the comparison of theoretical predictive model with experimental data, where theoretical predictive values of thermal conductivity are presented. For Equation 1, *p* is the aspect ratio of CuO microdisks, and the average value is 20 obtained by analyzing SEM images of CuO microdisks. *d* is the thickness of CuO microdisks (90 nm). *k*_f_ is the thermal conductivity of CuO microdisks (33 W/mK [34,35]). After trial and error analyses, *R*_k_ is set to 1.2 × 10^−7^ Km^2^/W. It can be seen that the experimental thermal conductivity data of thermal grease with CuO microdisks are in reasonable agreement with the predicted results of Equation 1. For Equation 3, *p* (1), *d* (1 μm), and *k*_f_ (33 W/mK) are aspect ratio, diameter, and thermal conductivity of CuO microspheres, respectively. Its predictive curve is also in reasonable agreement with the experimental thermal conductivity of thermal grease with CuO microspheres. However, for CuO nanoblocks, *p*, *d*, and *k*_f_ are, respectively, equal to 3, 130 nm, and 33 W/mK, and the predicted results of Equation 2 deviate from the experimental values. It is postulated that the nanosized CuO blocks could form many clusters in the silicone base, which could increase the actual size of CuO particles. Therefore, the aspect ratio of CuO nanoblocks will increase. At the same time, the clusters are favorable for thermal conductivity enhancement of thermal grease [30]. Add it all up and the shape factor has a great influence on thermal conductivity improvement of thermal greases with different CuO structures. The larger the shape factor is, the stronger the ability to thermal conductivity augment is. Interpreting this increased thermal conductivity as a high aspect ratio, it is reasonable to increase the particle-to-particle contact and the corresponding geometric parameter [26,27,36]. This structures some effective thermal conductive networks, in turn facilitating phonon transfer and resulting in high enhancement in thermal conductivity.

## Conclusions

In this work, we synthesized three kinds of CuO structures, namely, CuO microdisks, CuO nanoblocks, and CuO microspheres, by solution-based synthetic methods. The morphologies and crystal structures of these CuO structures were analyzed by SEM images and XRD patterns, respectively. A remarkable thermal conductivity improvement of thermal greases containing these CuO structures was obtained. The thermal conductivity of thermal greases increased with increasing loading of CuO structures and decreased slightly as temperature went up. Meanwhile, the thermal conductivity enhancement of thermal grease with CuO microdisks was higher than that of thermal greases with CuO nanoblocks and CuO microspheres. The comparison of Nan's theoretical predictive model with experimental data indicated that the shape factor had a great influence on thermal conductivity improvement of thermal greases with different CuO structures. CuO microdisks with large aspect ratio could increase the particle-to-particle contact, thus formed some effective thermal conductive networks, in turn resulting in high thermal conductivity enhancement.
